# Metabolic inequality in microbial communities

**DOI:** 10.64898/2026.04.14.718602

**Published:** 2026-04-17

**Authors:** Emmi A. Mueller, Jay T. Lennon

**Affiliations:** 1 Department of Biology, Indiana University, Bloomington, IN 47405, USA

**Keywords:** metabolic theory, phenotypic heterogeneity, ecology, scaling, productivity

## Abstract

How metabolic activity is distributed among individuals determines the scaling of cellular physiology to higher levels of biological organization. Yet the mechanisms that generate this heterogeneity and shape its distribution remain largely unresolved. We quantified single-cell metabolism in microbial communities spanning aquatic, terrestrial, and host-associated ecosystems. Across more than one million cells, metabolic activity followed a long-tailed distribution best described by a lognormal model, with a small subset of individuals contributing disproportionately to community metabolism. In some cases, the most active 20% of cells accounted for over 90% of metabolic output, although this pattern became less pronounced in more productive environments. To assess the consequences of metabolic inequality, we developed a model linking single-cell activity to community respiration. Because respiration responds nonlinearly to enzyme activity, variation among cells does not translate proportionally into ecosystem-level fluxes. As a result, ignoring metabolic heterogeneity can bias estimates of community respiration by up to 60%. Our findings reveal a general pattern of metabolic inequality across microbial communities in disparate habitats. Accounting for this structure is critical for understanding how microorganisms shape ecosystem processes and for improving predictions of large-scale biogeochemical dynamics.

**Significance:**

Inequality is a common feature of social, economic, and physical systems. It also arises in nature, where a small fraction of individuals accounts for an outsized share of biological output, including reproduction, immunity, and diversity. Here, we show that metabolic activity in microbial communities follows a characteristic long-tailed distribution that consistently emerges across diverse ecosystems, including lakes, soils, ocean plankton, marine sediments, and mammalian guts. Rather than a ‘rich-get-richer’ dynamic, metabolism becomes more evenly distributed among individuals in more productive environments. An explicit representation of metabolic inequality can improve predictions of how microbial communities, and the processes they support, respond to environmental change.

Metabolism is the sum of life’s chemical reactions and forms a unifying foundation for ecological theory, linking structure and function across all scales and levels of biological organization [1-4]. While metabolic rates vary widely among species and underpin many ecological predictions [5-8], they are also highly plastic, responding to abiotic and biotic conditions that fluctuate across space and time [9-11]. Even among genetically identical individuals maintained under highly controlled conditions, stochastic processes can introduce variability that influences metabolic output [12, 13].

Among living systems, microorganisms are particularly well-suited for examining individual-level metabolic variation. They encode a vast repertoire of catalytic functions [14-16], but their contributions to ecosystem processes depend not only on which taxa are present but also on physiological differences among individual cells [17, 18]. This variation challenges mean-field assumptions and makes microbial communities ideal for investigating how individual traits scale to influence collective outcomes. For example, many metabolic tasks rely on cooperative interactions that no single cell can perform alone [19]. Such interactions give rise to metabolic networks that enable microbes to thrive in diverse habitats and drive transformations essential to the stability of ecosystems, from host microbiomes to the biosphere [20, 21]. As a result, microbial traits and physiological properties are increasingly being integrated into models that aim to improve predictions of Earth system processes [22-26]. Yet these models often overlook the causes and consequences of metabolic variation among individual cells, reducing ecological dynamics to averages that mask underlying heterogeneity [27].

Without understanding how metabolic activity is distributed among community members, our ability to forecast how microorganisms contribute to ecosystem processes remains limited. Many models simplify this heterogeneity by assuming that all individuals contribute equally, yielding a uniform distribution in which each of the *N* individuals in a community accounts for 1/*N* of the total activity (*A_total_*) [28, 29]. Even when models explicitly account for variation in metabolism, for instance with a Gaussian distribution, the underlying assumption remains one of symmetry around a mean value (*A_total_/N*). However, complex assemblages of microorganisms are unlikely to exhibit such equality. Most cells do not operate at their full metabolic potential, and many can persist in an inactive state for extended periods of time [30]. If cells effectively exist in on/off states, activity may follow a bimodal distribution. If instead activity spans a physiological continuum, long-tailed distributions may emerge, consistent with the Pareto principle, in which a small fraction of individuals accounts for a disproportionately large share of total output [31]. Skewed distributions such as the lognormal, gamma, or power-law can arise from additive, multiplicative, or stochastic processes that govern key metabolic functions, including gene expression, biomass accumulation, and variation in growth rate [32-35].

Determining whether microbial communities exhibit a characteristic distribution of activity is essential for developing a general theory of metabolic ecology, which seeks to explain how energy and metabolism structure biological organization across scales [2]. A model that captures both the central tendency and the degree of inequality in activity (Fig. 1) provides a basis for investigating factors that influence metabolic organization, including growth strategies, functional traits, and resource availability. Because productivity governs energy and resource supply, it likely influences the distribution of metabolic activity within communities [8, 36]. For example, if productivity disproportionately enhances resource access for already active individuals, metabolic distributions may become more skewed, consistent with preferential attachment dynamics [37, 38]. Alternatively, if productivity broadens access across individuals, activity may become more evenly distributed [39]. Taken together, shifts in the shape of metabolic distributions along environmental gradients can reveal underlying constraints and help identify general principles that govern microbial metabolism across ecosystems.

In this study, we quantified single-cell activity across a range of environments and growth conditions to examine how metabolism is distributed among individuals belonging to diverse bacterial species and complex microbial communities. Using flow cytometry to quantify reductase activity associated with the electron transport chain, we characterized the distribution of metabolic activity in freshwater microbiomes and examined how its shape changed with growth regimes and productivity. To assess the generality of these patterns, we compared them to metabolic activity distributions observed in terrestrial, marine, and host-associated communities. Finally, we develop a model to examine the consequences of metabolic inequality and its potential to bias estimates of ecosystem processes. Together, these approaches provide a framework for linking patterns of metabolic organization to ecological theory and Earth system forecasting.

## Results and Discussion

Across a wide range of growth conditions and ecosystems, metabolism was unevenly distributed within microbial communities, with many cells exhibiting low metabolic activity and relatively few exhibiting high activity. The resulting distributions were consistently right-skewed and best described by a lognormal model. Using a diverse collection of bacterial isolates, we found that this pattern was conserved across taxa, indicating that the lognormal distribution does not arise from aggregation of distinct species-level distributions. Notably, skewness declined with increasing productivity, suggesting that greater resource availability enables more cells to sustain active metabolism. To test the generality of these patterns, we analyzed single-cell data from both laboratory and environmental samples spanning freshwater, marine, soil, and gut ecosystems. Applying the principles of nonlinear averaging, we show that accounting for skewed activity distributions yields more accurate and mechanistically grounded estimates of aggregate metabolic processes, with the potential to improve forecasts of ecosystem-scale dynamics.

### 1. Metabolic activity is unequally distributed

Uneven distributions of activity are more likely than uniform ones. There are simply many more ways for activity to be unevenly distributed among individuals [40]. As a result, heterogeneity emerges as the most probable outcome, even in the absence of specific ecological or physiological drivers. In many complex systems, this tendency gives rise to inequality, in which a small fraction of individuals accounts for most of the total output. Such uneven distributions may follow the Pareto principle, where roughly 80% of output is generated by 20% of producers. To test whether microbial communities exhibit such patterns, we quantified the fraction of activity attributed to the top 20% of cells across three contrasting growth regimes: (1) batch cultures, where resource depletion, biomass production, and waste accumulation drive transitions through lag, exponential, and stationary phases; (2) continuous culture (chemostats), where dilution rate determines growth at steady state through the balance of resource supply and washout; and (3) environmental samples, where cells were collected in a freshwater reservoir along a hydrological flow path that establishes a gradient of productivity.

Metabolic inequality was pronounced across growth regimes, with the top 20% of individuals contributing over 90% of total activity in some cases (Fig. 2). Batch culture exhibited the greatest variability (Fig. 3). In lag phase, when resources were replete, about 75% of total activity was produced by the 20% most active cells (Fig. 3B, S1, & S2). As cultures transitioned into the exponential and stationary phases, activity became more evenly distributed (32–41%), yet the most active individuals still contributed about 1.5 times their expected share of total activity (Fig. 3C-D, S1, & S2). In contrast to batch cultures, environmental samples and continuous cultures exhibited narrower ranges, with 51–90% and 66–91% of total metabolism attributable to the most active cells, respectively (Fig. 2). Although inequality varied across growth phases, metabolic activity remained consistently skewed, with a subset of cells persisting in a low-activity state even under optimal conditions. This pattern is consistent with bet-hedging strategies, in which some individuals enter a quiescent state that reduces instantaneous fitness but buffers populations against environmental change [41-43]. More broadly, it highlights the limitations of treating microbial communities as homogeneous black boxes, which can mask the disproportionate contributions of individual cells to ecosystem processes [44, 45].

### 2. A characteristic distribution for metabolic activity

Identifying how activity is distributed among individuals is essential for understanding the causes and consequences of phenotypic heterogeneity, advancing metabolic theory, and improving our broader understanding of functional ecology [46]. Yet, no widely accepted model captures how metabolism is partitioned or how resulting distributions shift across environmental gradients and ecosystem types. Parameterizing a distributional model provides a concise way to summarize complex datasets while also establishing a common framework for cross-study comparisons. Moreover, these models can serve as powerful tools for ruling out implausible explanations, generating testable hypotheses, and providing baselines for assessing how populations, communities, and ecosystems respond to perturbations.

**Figure 1.**
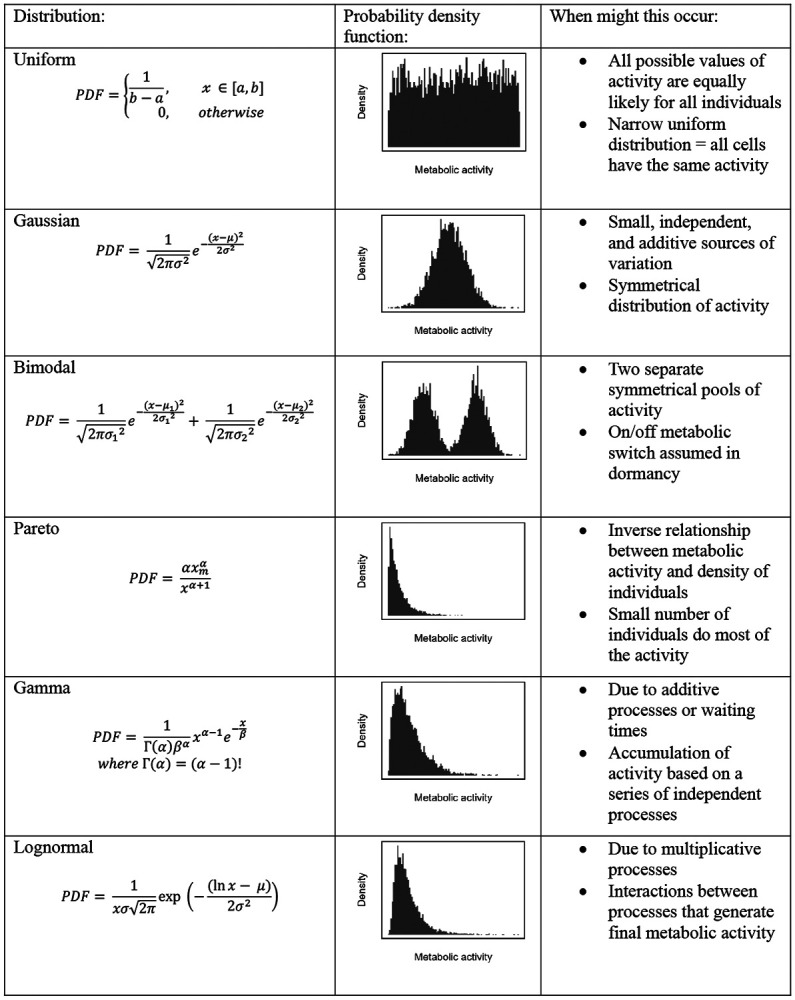
Metabolic distributions. Potential distributions of metabolic activity at the single-cell level and the mechanisms that generate them. Each row shows the probability density function (PDF), its visualization, and a summary of potential mechanisms and their implications for community-level metabolic activity.

Using over 500,000 single-cell reductase measurements, we evaluated whether a single distribution could reliably describe the metabolic activity of aquatic microorganisms under different growth conditions. Consistently, the lognormal distribution was the best-fitting model, outperforming all others (uniform, bimodal, Gaussian, gamma, and Pareto; see (Fig. 1) based on both *r*^2^ values (Fig. 4A; Table S3 & S4; Batch: *F*_3_ = 84.91, *P* < 0.0001; Continuous: *F*_3_ = 449.4, *P* < 0.0001; Environmental: *F*_3_ = 1588, *P* < 0.0001) and Kolmogorov-Smirnov (K-S) statistics (Fig. 4B; Table S3 & S5; Batch: *F*_3_ = 55.2, _P_ < 0.0001; Continuous: *F*_3_ = 803, _P_ < 0.0001; Environmental: *F*_3_ = 1065, *P* < 0.0001). According to Akaike Information Criterion (AIC) and Bayesian Information Criterion (BIC), the lognormal provided the best fit for all continuous-culture and environmental samples tested (28 of 28 and 36 of 36, respectively). In batch cultures, the best-fitting models were more variable: the lognormal ranked best in 7 of 50 samples, compared to 16 for the gamma, 25 for the Gaussian, and 2 for the Pareto (Fig. S3). Bootstrapped *r_m_*^2^ values and quantile–quantile (Q–Q) plots further supported the lognormal model, which achieved *r_m_*^2^ = 0.91 compared with 0.83 for the gamma 0.72 for the Gaussian, and 0.80 for the Pareto (Fig. 4C-F, SI Appendix). Together, these results demonstrate that metabolic activity is strongly right skewed, indicating that a small subset of individuals is substantially more active than the rest of the sampled cells.

The robustness of the lognormal model in describing reductase activity provides insight into the potential mechanisms underlying the pattern of microbial inequality. Specifically, it allowed us eliminate some a *priori* models that fit the data poorly (Fig. 1). For example, the bimodal model failed to capture activity patterns, suggesting that metabolism in complex communities cannot be explained by simple on/off regulation, while the Gaussian model, which assumes additive, independent effects around a mean, also performed poorly. The gamma model, which would suggest that metabolism is the sum of sequential stochastic processes with exponential waiting times, provided a better fit by capturing right-skew in activity [47]. However, the Pareto and lognormal models fit best overall.

The distinction between these best-fitting models provides insight into the mechanisms generating metabolic inequality. While both capture right-skewed patterns, they differ in their interpretation. The Pareto should arise from unbounded reinforcing feedbacks that produce heavy-tailed distributions, whereas the lognormal arises from stochastic proportional changes around an equilibrium, generating finite variance and lighter tails [31]. The lognormal therefore represents the most probable, high-entropy configuration of a multiplicative system in which a small subset of cells contributes disproportionately to total metabolism [48, 49]. Its generality also explains why the lognormal commonly arises in diverse biological contexts, including transcript levels [50], body sizes [51], species abundances [52], and cell growth dynamics [35].

### 3. Taxonomic and phylogenetic contributions to metabolic inequality

Microbial communities are made up of species with distinct growth strategies and functional traits. As a result, the community-level distribution of metabolic activity must reflect those of its constituent taxa and thus could arise from the aggregation of distinct species-level distributions. To evaluate this hypothesis, we measured single-cell reductase activity in a diverse set of heterotrophic bacteria grown under oligotrophic conditions. Across all species, metabolic activity followed a lognormal distribution, indicating that the community-level pattern is unlikely to result from aggregating fundamentally different species-level distributions. Nevertheless, the degree of metabolic inequality varied among species, even under identical growth conditions (Fig. S4). Most species exhibited moderate skew, quantified by the log-scale standard deviation (σ) of the fitted lognormal distribution. A subset, however, showed pronounced inequality, with the most active 20% of cells accounting for more than 70% of total activity (Fig. S4). Notably, variation in σ exhibited a strong phylogenetic signal consistent with Brownian motion (Pagel’s λ = 0.99, *P* < 0.0001), indicating that evolutionary divergence shapes metabolic inequality. Thus, the lognormal form is shared across species, but taxon-specific differences in the degree of metabolic inequality may influence the community-level distribution.

### 4. Metabolism is more evenly distributed in productive environments

Productivity is the rate at which energy is converted into new biomass. It sets a fundamental constraint on the amount of biological work that can be performed and, as a result, shapes patterns of abundance, diversity, body size, species interactions, and ecosystem functioning [53]. Thus, we hypothesized that productivity should influence the distribution of metabolic activity among individuals within a community. On the one hand, increased productivity could disproportionately fuel already active individuals, thereby amplifying metabolic inequality. On the other hand, increased productivity could elevate activity across individuals while reducing differences among them, resulting in a more even metabolic distribution.

To test these contrasting scenarios, we examined the relationship between bacterial productivity (BP) and metabolic inequality across two complementary datasets. In a freshwater reservoir, where BP varied longitudinally along the hydrological flow path (Fig. S5), metabolic skew (σ) declined with increasing productivity (Fig. 5). Similarly, in laboratory chemostats, experimental manipulation of BP in communities derived from the freshwater reservoir (Fig. S6) led to a decline in metabolic skew (Fig. 5). Using an indicator-variable multiple regression, we found that the baseline skew, represented by the intercept, was approximately twofold higher in chemostats than in the reservoir ecosystem (*R*^2^ = 0.96, *F*_3,60_ = 488.5, *P* < 0.0001, Table S6). However, the slopes relating skew to log_10_(BP) were statistically indistinguishable (−0.14 ± 0.031), indicating that metabolic activity becomes more evenly distributed with increasing productivity in aquatic microbial communities.

Our findings run counter to the common aphorism that “the rich get richer and the poor get poorer.” One possible explanation is that low-activity individuals invest more in acquiring resources as availability increases, because the marginal gains are greater. Alternatively, highly active individuals may experience diminishing returns under resource-rich conditions, particularly if uptake or storage capacity becomes saturated. Our results are also consistent with observations from non-biological systems, such as economies and hierarchical organizations, where resource additions benefit a larger fraction of the population rather than a select few [37]. Together, these results suggest that increased resource availability may promote more equitable distributions of output across complex systems, rather than reinforcing existing inequalities.

### 5. Cross-ecosystem consistency in metabolic inequality

To evaluate the generality of metabolic inequality, we examined its prevalence across diverse ecosystems. In oceans [54-57], lakes (Fig. 4), soils [58], sediments [59, 60], and guts [61], we found that the most active 20% of individuals accounted for 38% to 90% of total metabolic activity, consistent with Pareto-like dynamics (Fig. S9). Single-cell activity distributions were best described by the lognormal (Fig. S8, Table S3), outperforming alternative models regardless of ecosystem type. Notably, these outcomes were robust to differences in how cellular metabolism was quantified, including microautoradiography [54, 55, 57], DNA- and amino acid-based click chemistry [56-58], redox indicators [59, 60], and related approaches [61]. Together, our results indicate that metabolic inequality is widespread and follows a consistent, quantitatively structured pattern across ecosystems.

We next asked how much of the variation in metabolic inequality can be attributed to ecosystem type, given contrasts in environmental conditions and community composition across diverse habitats. Using the intraclass correlation coefficient from a random-intercept model to partition variance in the lognormal skew parameter (σ), we found that ecosystem identity explained 43% of the total variance (Fig. 6). The datasets analyzed here span ecosystems with wide ranges of redox state, temperature, pressure, solar input, pH, and modes of energy and carbon acquisition. Yet across these distinct habitats, from loamy soils to deep ocean sediments, we observe overlapping ranges of σ, indicating that ecosystem-specific signatures do not override broader patterns of metabolic inequality.

**Figure 2.**
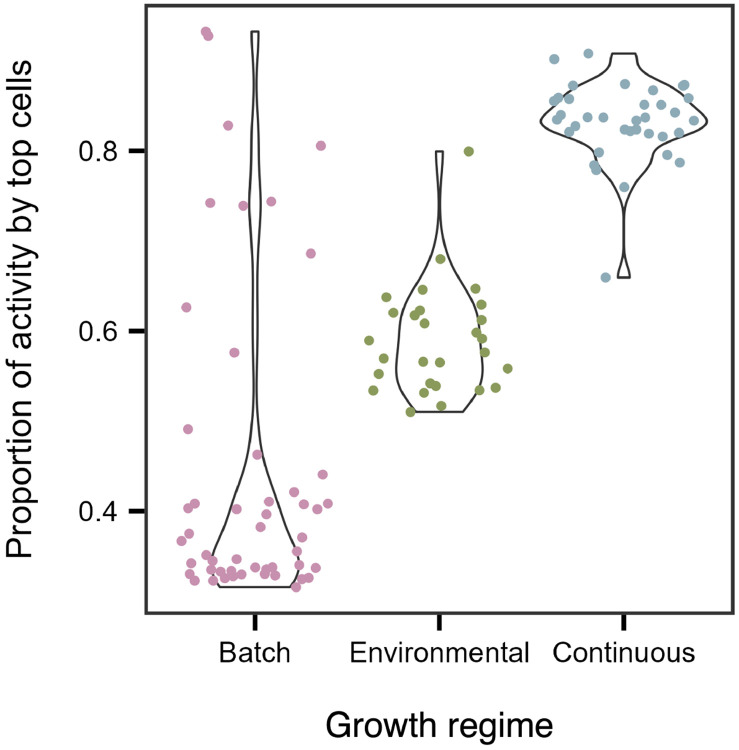
Metabolic activity is unevenly distributed. Violin plots showing that the 20% most active cells contribute a large but variable fraction of total community-level activity. Batch cultures exhibit the widest range, consistent with shifts in metabolic distributions across growth phases (lag, exponential, and stationary). In contrast, environmental samples and continuous cultures (chemostats) show narrower ranges.

**Figure 3.**
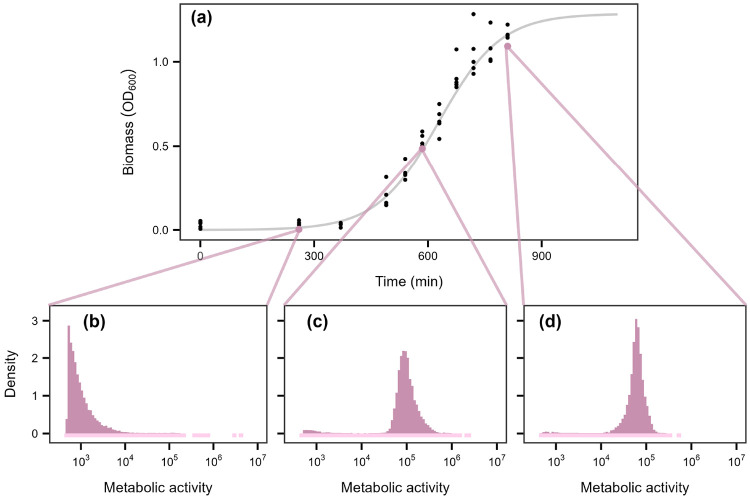
Growth-phase dependent shifts in metabolic activity. **(a)** Microbial biomass of a lake water community grown in batch culture over time. In the lower panels **(b-d)**, metabolic activity is reported as relative fluorescent units (RFU). Density plots show the distribution of metabolic activity across three growth stages: lag, exponential, and stationary. To highlight the tails of the distribution, low-density histogram bins are extended below zero using a lighter tint of the histogram color. **(b)** In lag phase, metabolic activity was low and highly right-skewed, with most cells exhibiting activity values below 10^5^ (median = 810; mean = 2,461). **(c)** In exponential phase, metabolic activity increased by approximately two orders of magnitude, and the distribution remained right-skewed (median = 93,083; mean = 111,569). **(d)** In stationary phase, the distribution became more even, with activity levels similar to those in exponential phase (median = 60,734; mean = 63,203).

More than half of the variation in skewness occurred within ecosystems, indicating that metabolic inequality is strongly influenced by habitat-specific conditions. For example, within marine systems, σ varied systematically between open-ocean and coastal habitats, while in gut-associated communities it differed between attached and free-living assemblages. Such patterns suggest that habitat structure, lifestyle, and productivity (Fig. 5) modulate the magnitude of inequality within ecosystems. Temporal dynamics likely amplify these effects. In our experiments, some of the largest shifts in σ reflected expansions and contractions in growth and cell division driven by fluctuations in resource availability (Figs. 3, 2). Because such dynamics are widespread across natural, engineered, and host-associated systems, they may represent a broadly important mechanism underlying variation in metabolic inequality.

### 6. Consequences of metabolic inequality for ecosystem functioning

We have shown that metabolic activity is unevenly distributed among cells across diverse habitats and environmental gradients (Fig. 6). However, understanding how this physiological variation influences ecosystem processes such as respiration and primary productivity remains a major challenge. Many relationships between traits and performance are inherently nonlinear, such that variation among individuals does not translate proportionally into system-level outcomes. For example, photosynthesis saturates with light, growth plateaus with increasing nutrient supply, and fitness often follows curved trait landscapes. When such nonlinearities are present, the performance predicted from the average trait value differs from the average performance across individuals. This outcome follows from Jensen’s inequality, which states that for nonlinear functions, f(E[x])≠E[f(x)], where E denotes the expectation (mean) of a trait x across individuals. Consequently, model predictions based on mean trait values can be biased.

**Figure 4.**
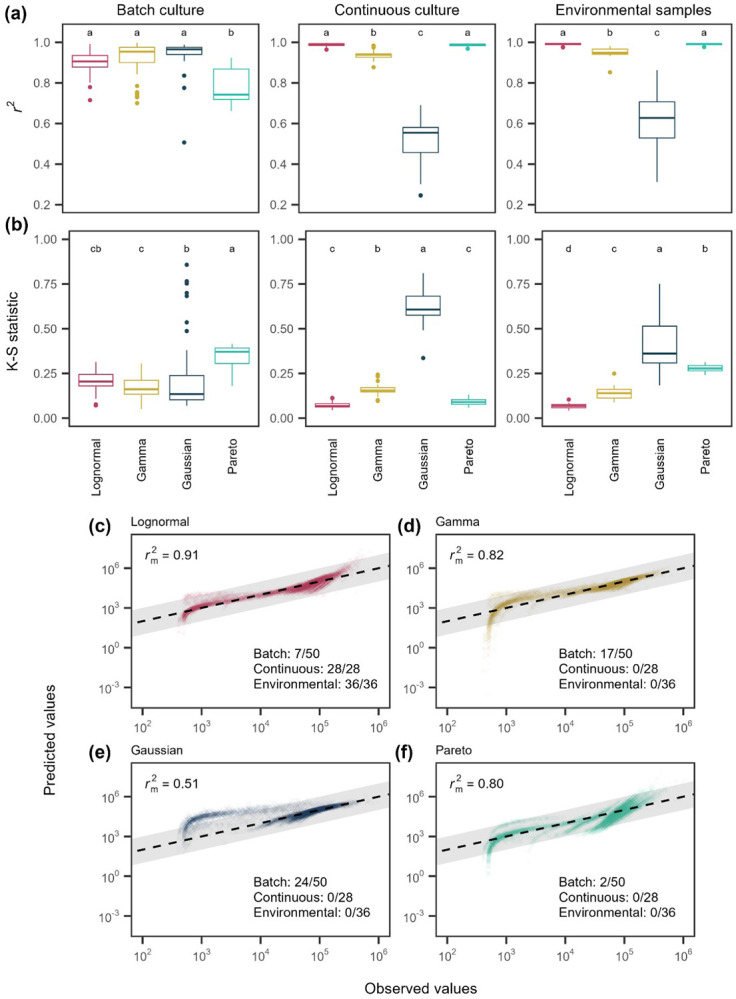
Model fits to single-cell metabolic activity distributions. Metabolic activity distributions for microbial communities across three growth regimes (batch culture, continuous culture, environmental samples) were fit with four candidate models: lognormal, gamma, Gaussian, and Pareto. **(A)** Coefficients of determination (*r*^2^) for each sample–model fit; letters denote groupings based on post hoc Tukey tests. **(B)** Kolmogorov–Smirnov (K–S) statistics, where lower values indicate better fit. **(C–F)** Quantile–quantile plots comparing observed and predicted activity for the lognormal, gamma, Gaussian, and Pareto models, respectively. Points represent 10,000 subsampled individuals across growth conditions. Solid lines indicate the 1:1 relationship, and shaded regions represent ±1 order of magnitude in activity. Numbers indicate the number of samples for which each model was the best fit, based on ΔAIC and ΔBIC > 2. Across metrics and conditions, the lognormal model provides the best overall fit among candidate models.

Although the magnitude of this bias depends on the curvature of the response function and the variance among individuals [62], it may also be influenced by higher-order features of the trait distribution, such as skewness, which often co-vary with variance. We explored how the lognormal distribution of single-cell reductase activity (Fig. 4) influences total respiration at the community level. We modeled cellular respiration (*R*) using the canonical Michaelis-Menten equation to represent the hyperbolic response of respiration to the per-cell level of reductase activity (*E*): $R=\left(R_{max}\times E\right)∕(K_{m}+E)$, where *R_max_* is the maximum per-cell respiration rate and *K_m_* is the half-saturation constant, representing the activity level at which *R* equals one half of *R_max_*.

**Figure 5.**
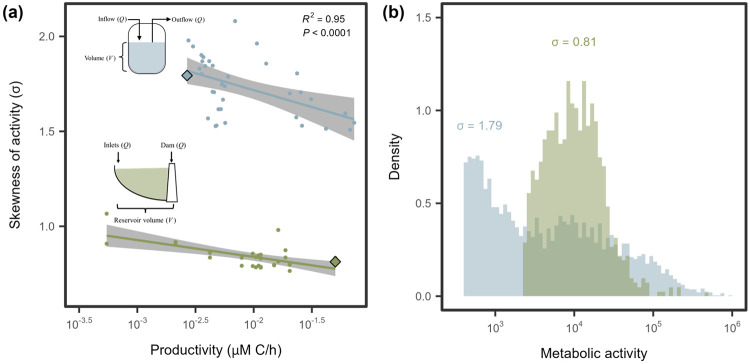
Productivity reduces metabolic inequality. **(A)** In both continuous culture (blue) and environmental samples (green), metabolic activity became less skewed with increasing productivity. Lines and shaded regions represent linear regression fits and 95% confidence intervals, respectively (Table S6). **(B)** Two representative samples, highlighted by diamond-shaped symbols in **(A)**, illustrate contrasts in distribution shape. While both are best fit by the lognormal model, the more skewed sample (blue, *σ* = 1.79) from a continuous culture with a low dilution rate shows a broader range of metabolic activity, with a pronounced tail extending to ~ 10^6^ RFU. The less skewed sample (green, *σ* = 0.81) from a reservoir site furthest from the dam outlet shows a more even distribution, with values extending only to ~ 10^5^ .

**Figure 6.**
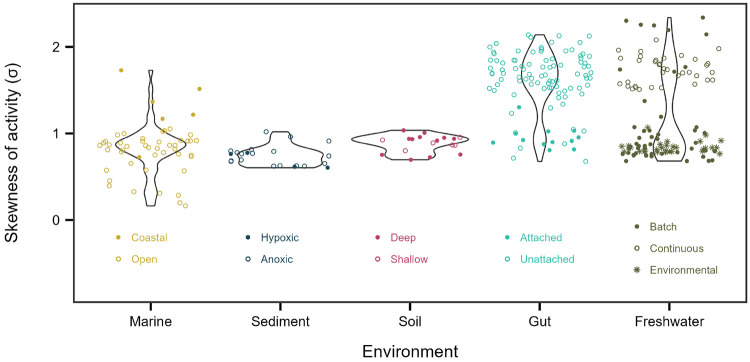
Metabolic inequality across ecosystems. Each point in a violin plot represents the skew in metabolic activity among individuals within a community, measured by the *σ* parameter from lognormal fits.

**Figure 7.**
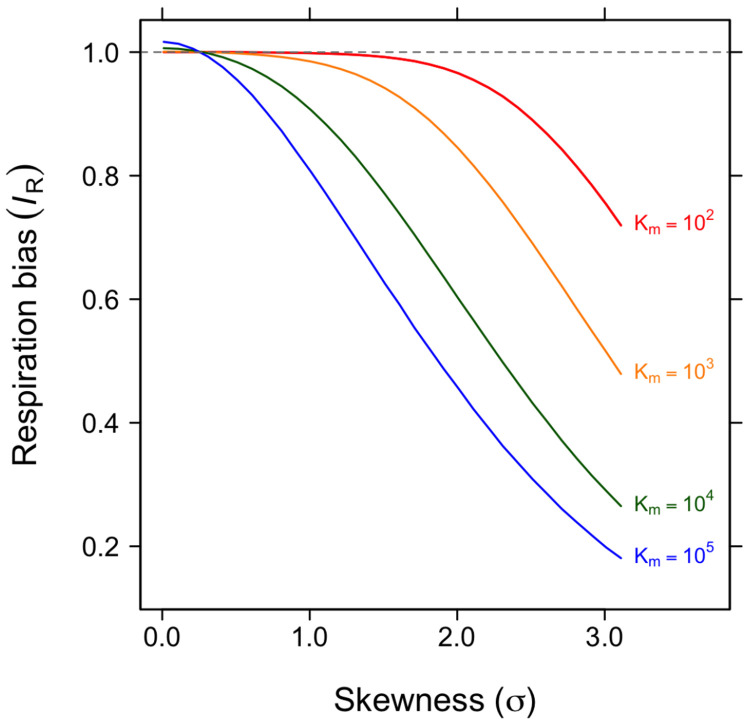
Consequences of metabolic inequality. Simulations show how the index of respiration bias (*I_R_*) varies with the skewness (*σ*) of lognormally distributed metabolic activity. *I_R_* is defined as the ratio of total community respiration predicted under a Gaussian distribution to that under a lognormal distribution with the same mean. Single-cell respiration is modeled using a Michaelis–Menten response to reductase activity (*E*), and community respiration is obtained by summing across cells. Each curve corresponds to a different value of the half-saturation constant (*K_m_*), which governs the degree of nonlinearity in the functional response. Bias is greatest at high skew and low *K_m_* , where individual cells reach *R_max_* at low levels of *E* , amplifying the effects of metabolic inequality.

To evaluate the effects of metabolic inequality on community respiration, we developed an index of respiration bias (*I_R_*). This index compares total respiration predicted under a lognormal distribution of reductase activity (*E*) with that predicted under a Gaussian distribution having the same mean. When *I_R_* = 1, aggregate respiration is equivalent under Gaussian and lognormal distributions of *E*, indicating no net effect of distributional shape on the aggregated response. Simulations show that respiration bias increases with the skewness (σ) of the lognormal distribution and is amplified when the functional response saturates more gradually at higher values of *K_m_* (Fig. 7). Under empirically observed levels of skewness (Figs. 5, 6), we estimate that community respiration could be overestimated by as much as 60%.

Efforts to improve ecosystem and Earth system forecasts increasingly aim to incorporate greater biological realism, particularly where feedbacks may arise [26, 63]. To date, much of this effort has focused on microbial traits related to nutrient acquisition, enzymatic activity, thermal sensitivity, and carbon use efficiency [64, 65]. However, these approaches rarely include explicit representations of metabolic heterogeneity within microbial biomass pools [66, 67]. Instead, most models treat individuals as physiologically identical under a common set of environmental conditions. Our results suggest that this simplification may overlook a fundamental feature of microbial communities. Incorporating skewness into even a simple model of microbial respiration reveals that metabolic inequality can substantially alter predicted ecosystem fluxes (Fig. 7). Together, these findings underscore the need to move beyond homogeneous biomass pools and explicitly represent individual-level variation in metabolic activity. Such representation could be achieved through agent-based models that allow distributional structure to emerge dynamically, or by incorporating parameterized activity distributions into trait-based and ecosystem models to evaluate how metabolic inequality propagates through trophic levels and food webs [68, 69]. Extending these approaches to Earth system models would enable assessment of how microbial inequality influences large-scale biogeochemical dynamics and climate–carbon cycle feedbacks.

## Conclusion

Inequality among individuals is a recurring feature of complex systems, spanning economies, social networks, and physical processes. Here, we show that metabolic activity within microbial communities is likewise organized according to a shared underlying structure. Across growth regimes, taxa, and ecosystems, activity distributions were uniformly right-skewed and best described by a lognormal model, with a minority of cells accounting for a disproportionate fraction of total function. Although the magnitude of inequality shifts with productivity, habitat structure, and temporal dynamics, the widespread occurrence of this distribution points to a fundamental organizing principle of microbial community metabolism. By extension, these findings suggest that metabolic inequality may shape species interactions and community stability: if highly active individuals are more likely to be grazed by predators, infected by viruses, or engage in syntrophic exchange, then activity may be concentrated within a subset of the population, focusing ecological risk and interaction strength with consequences for resilience and coexistence. Similar long-tailed distributions arise across ecosystems and taxa, suggesting that inequality is an emergent and perhaps unavoidable consequence of multiplicative biological processes. Recognizing and quantifying these inequalities provides a foundation for linking individual-level variation to community dynamics, ecosystem functioning, and the broader principles governing complex adaptive systems.

## Methods

### Growth regimes—

To investigate how metabolic activity is distributed among individuals, we sampled complex multispecies communities under different growth regimes. First, we propagated a freshwater microbial community under batch-culture conditions to determine how the distribution of metabolic activity changes over a growth cycle. We inoculated 1 mL of lake water from Goodman Lake, a meso-eutrophic surface mine lake in Greene-Sullivan State Park, Indiana, USA (39.013 °N, 87.236 °W) into replicate (*n* = 5) Erlenmeyer flasks (25 mL) containing LB medium (4 mL), which were then incubated on a shaker table (200 rpm) at 37 °C. At 45-min intervals, we measured biomass via optical density (OD_600_ nm) using a BioPhotometer plus (Eppendorf, Hamburg, Germany) and took samples for flow cytometry as described below. To characterize the growth curve dynamics, we fit optical density (OD_600_ nm) measurements with a logistic growth model using the ‘nls’ function (‘stats’ R package, v. 4.3.2).

Second, to determine how the distribution of metabolic activity changes with community growth rate, we manipulated the dilution rate in a set of continuous flow reactors (*n* = 36). We inoculated each of these chemostats (40 mL) with microorganisms from University Lake, a meso-eutrophic reservoir located in Griffy Woods, Bloomington, Indiana, USA (39.189 °N, 86.503 °W; [70]). Using a combination of peristaltic pumps and manual pipetting, we delivered twice-autoclaved lake water as growth medium and established dilution rates spanning over seven orders of magnitude (10^−6.5^ to 10^0.5^ h). As a source of immigration, we added a dilute suspension of lake sediment daily, corresponding to 1% of the estimated total cell turnover [71]. After 20 d, we destructively harvested each chemostat, measured bacterial productivity (BP) using a tritiated leucine incorporation assay [72], and collected samples for flow cytometry as described below.

Third, to determine the distribution of metabolic activity of microorganisms in a natural ecosystem, we obtained water samples from University Lake, which is a small (6.5 ha) reservoir exhibiting predictable spatial patterns along hydrological flow paths, where gradients in nutrient and light availability shape microbial diversity and productivity [70, 73]. Samples were collected every 25 m from the stream inlet to the dam. Upon return to the laboratory, we measured bacterial productivity (BP) using a tritiated leucine incorporation assay [72] and collected samples for flow cytometry as described below.

### Single-cell metabolic activity—

We measured single-cell metabolism using BacLight^™^RedoxSensor^™^Green (RSG; excitation/emission: 490/520, Invitrogen), a fluorescent probe that emits stable green fluorescence when reduced by bacterial reductases. These enzymes play a central role in cellular respiration, catalyzing redox reactions in which electrons are transferred to terminal electron acceptors, thereby generating proton gradients and contributing to ATP production. Because RSG responds broadly to redox activity rather than targeting a specific reductase, it serves as a general indicator of metabolic activity under both aerobic and anaerobic conditions. Previous studies have shown that RSG fluorescence is positively correlated with bacterial activity and viability across diverse taxa and environments [59, 74, 75]. To stain samples, we diluted each sample 1:100 in phosphate buffered saline (pH = 7.4) and added 1 *μ*L RSG to 1 mL of the diluted sample. We then incubated the samples at 37 *°*C in complete darkness for 15 min. To preserve fluorescence for analysis, we fixed the cells by adding 25 *μ*L of 25% glutaraldehyde and incubated the samples for an additional 30 min at 25 °C in the dark. Samples were stored at 4 °C until they were run on a NovoCyte 3000 flow cytometer (Agilent, Santa Clara, CA, USA) equipped with a 50 mW laser-emitting light. RSG fluorescence was captured with the 488 nm laser using a 530/30 nm filter. For each sample, we collected 10,000 events with a forward scatter height (FSC-H) greater than 300 (arbitrary units), using a gain of 453 and a flow rate of 14.7 *μ*L min^−1^. These settings minimized noise from small FSC and SSC values and ensured that the event rate remained below 1,000 events per second, reducing the likelihood of coincident cell detection.

Once the flow cytometry events were collected, we identified live cells using electronic gating with NovoExpress (NovoCyte software, v. 1.4.1). We first gated for single-cells using a side-scatter height vs. area (SSC-H vs. SSC-A) plot to exclude aggregated cells and instrument noise (Fig. S10A). Next, we removed background signal by applying an RSG fluorescence versus event count gate based on cell-free PBS controls (Fig. S10B). To ensure that viable cells with low RSG fluorescence were not excluded, we validated this gate against unstained live cell samples. Flow cytometry data were then exported as .fcs files and imported into R using the Bioconductor ‘flowCore’ package (v. 2.14.1) for downstream analysis.

### Bacterial isolates and phylogenetic analyses—

We isolated bacteria from surface water collected at University Lake (see above) by plating dilutions onto R2A agar and incubating plates at 25 °C in the dark. All strains were purified through multiple transfers of single colonies before assessing single-cell metabolic activity using the staining and flow cytometric procedures described above, after harvesting cells following 24 h of growth in 1/10 R2A broth on a shaker (200 rpm) at 25 °C. We identified each strain by sequencing the 16S rRNA gene. Genomic DNA was extracted using a Microbial DNA Isolation Kit (MoBio), and the 16S rRNA gene was amplified using primers 8F and 1492R following established protocols [76]. Sequences were aligned using MAFFT with a high-accuracy global alignment strategy [77], and a maximum likelihood phylogeny was inferred using RAxML-NG (v1.2) under a GTR+Γ model of sequence evolution, with branch support assessed using 200 bootstrap replicates [78]. Finally, skewness (σ) of the lognormal fits was mapped onto the phylogeny using the ape package [79], and phylogenetic signal was assessed using Pagel’s λ with the phytools package [80] in R.

### Statistical comparison of model fits—

To assess the degree of metabolic inequality in a sample, we quantified the share of total activity contributed by the top 20% most active individuals. We then examined how this measure of skew varied across growth conditions in samples collected from batch cultures, continuous cultures (chemostats), and environmental systems. To characterize the underlying distributions of the metabolic data, we fitted six candidate models: uniform, bimodal, Gaussian, gamma, lognormal, and Pareto. For each sample, we fit the raw metabolic activity values using maximum likelihood estimation with the ‘mle2’ function from the ‘bbmle’ package [81] employing the ‘L-BFGS-B’ method (version 1.0.25). Given that metabolic activity cannot be negative, we used a truncated Gaussian with a lower bound of 0 and no upper bound. The probability distribution function, parameters, starting values, and parameter bounds for each candidate model are provided in Table S1.

We determined the best fitting model for each sample by comparing model performance using a suite of goodness-of-fit metrics. First, we calculated *r*^2^ values for each sample and used ANOVA followed by a *post hoc* Tukey test to determine the best model for each growth condition. Second, we calculated the Kolmogorov-Smirnov statistic (K-S; ‘ks.test’ function) to assess the agreement between the observed data and the distribution predicted by the fitted model. The statistic ranges from 0 (complete agreement) to 1 (no overlap between distributions). Third, for each sample we selected the best fitting model based on the lowest Akaike Information Criterion (AIC) and Bayesian Information Criterion (BIC) values. Models were considered meaningfully different when the absolute difference in information criterion scores (∣ΔIC∣) exceeded 2. We visualized model performance using quantile-quantile (Q-Q) plots of the observed metabolic activity against the predicted metabolic activity, subsampled to 10,000 cells per model. Last, to estimate an overall *r*^2^ value for each candidate model across all growth conditions, we randomly subsampled 5,000 cells from the full freshwater dataset (~ 600,000 cells) across 114 samples). We repeated this 10,000 times, calculating the *r*^2^ for predicted vs. observed values in each iteration and averaged the results to obtain a bootstrapped *r_m_*^2^ value.

### Productivity-inequality relationships—

We measured bacterial productivity (BP) by tracking the incorporation of 3H-leucine into microbial communities [72]. We spiked 1.5 mL samples with ^3^H-leucine for a final concentration of 50 nM. After incubating the samples for 1 h, we stopped the reactions by adding 300 *μ*L of 3 mM trichloroacetic acid (TCA). We then washed the cells with 0.3 mM TCA to remove any unincorporated radioactive leucine before covering the samples in scintillation fluid for measurement on a Tri-Carb 2100TR Liquid Scintillation Counter (instrument efficiency: 64.3%; Packard Instrument Company, Meriden, CT, USA). For each sample, we included a voucher replicate in which leucine incorporation was stopped immediately after the isotope spike to determine background radioactivity prior to biological uptake. We converted counts per minute (CPM) to disintegrations per minute (DPM) and used voucher-corrected values to estimate the amount of leucine incorporated. These values were then converted to rates of carbon assimilation (*μ*M C h^−1^) using estimates of the fraction of leucine in protein and cellular carbon per protein [82].

To evaluate how productivity influences the distribution of metabolic activity, we examined the relationship between bacterial productivity (BP) and the shape of metabolic activity in the two growth regimes where BP was measured (continuous culture and environmental samples). In the lognormal model, the σ parameter (standard deviation in log-space) reflects the degree of skew in metabolic activity. We tested the effect of BP on σ model fits using multiple regression with indicator variables [70, 83] where BP was treated as a continuous predictor variable and growth condition (i.e., continuous culture vs. environmental samples) as a categorical variable. An interaction term was included to test whether the relationship between BP and σ differed between growth conditions. All statistical analyses were conducted in R (v 4.3.2; R Core Team, 2024).

### Simulation model—

We developed a model to examine how the statistical distribution of enzyme activity levels among individual cells influences community-level respiration. For each simulation, we sampled 10^6^ cells with independently drawn enzyme levels. Baseline enzyme activity (*E*) was drawn from a Gaussian distribution $\left(\bar{x}=10^{5},\;\text{sd}=0.25\times \bar{x}\right)$, and respiration (*R*) for each cell was calculated according to the Michaelis-Menten function, where maximum respiration (*R_max_*) was standardized to 1 and half-saturation constants (*K_m_*) ranged from 10^2^ to 10^5^. To evaluate the effect of distributional skew, we replaced the Gaussian with lognormal activity distributions having the same arithmetic mean (10^5^) but varying log-space standard deviations (σ = 0.01 - 3.5). For each *K_m_*-σ combination, we computed the mean respiration across all cells and expressed it as a ratio relative to the Gaussian baseline, which we use as index of respiration bias (*I_R_*). Because total community respiration is the sum across individuals (i.e., proportional to the mean for fixed population size), this ratio also reflects differences in aggregate respiration. Ratios > 1 indicate that the lognormal distribution yields higher predicted respiration than the Gaussian baseline, whereas ratios < 1 indicate lower respiration. This approach captures the influence of skew (and associated changes in variance) while holding mean activity constant, allowing us to quantify how increasing inequality among cells alters predicted community-level respiration.

## Supplementary Material

Supplement 1
